# Bridging the Gap: Evaluating the Effectiveness of Haptic Simulators Compared to Traditional Methods in Preclinical Dental Education

**DOI:** 10.3390/dj14050314

**Published:** 2026-05-20

**Authors:** Pedro C. Lopes, Sara Lopes, Rute Rio, Hélder Costa, Adriana B. Matos, Nélio Veiga, Maria J. Correia

**Affiliations:** 1 Faculty of Dental Medicine, Universidade Católica Portuguesa, 3504-505 Viseu, Portugal; snlopes@ucp.pt (S.L.); rpsousa@ucp.pt (R.R.); hecosta@ucp.pt (H.C.); nveiga@ucp.pt (N.V.); mcorreia@ucp.pt (M.J.C.); 2 Center for Interdisciplinary Research in Health, Universidade Católica Portuguesa, 3504-505 Viseu, Portugal; bona@usp.br

**Keywords:** haptic simulator, dental education, virtual reality, psychomotor training, preclinical training, dentistry

## Abstract

**Background:** Haptic simulation technologies are increasingly integrated into preclinical dental education to support procedural skill development. However, the extent to which haptic simulators improve performance compared to traditional phantom-head-based training remains unclear. Our goal is to systematically evaluate the effectiveness of haptic simulators in operative dentistry training, compared with conventional approaches. **Methods:** A systematic literature search was conducted in PubMed, Scopus, and Cochrane (2015–2025), complemented by manual searching, to identify studies evaluating virtual reality haptic simulators in preclinical operative dentistry education. The search strategy, structured according to the PICO framework, included preclinical undergraduate dental students, interventions with virtual reality haptic simulators, comparisons with conventional methods, and objective assessment of technical performance. Relevant data were extracted in a standardized manner, and the methodological quality of randomized controlled trials was assessed using RoB 2.0, while non-randomized studies were evaluated using ROBINS-I v2. **Results:** Of the 66 identified articles, 5 studies were included. The use of virtual reality simulators with haptic feedback in preclinical dental students was associated with increased efficiency in cavity preparation, reflected by reduced execution time and improved learning curves, as well as specific technical gains such as depth control. Overall cavity preparation quality was comparable to that achieved with conventional methods, with virtual reality being well accepted as an effective complementary tool in preclinical operative dentistry education. **Conclusions:** Haptic simulators appear effective for early preclinical skill development in operative dentistry and may complement traditional instruction. Evidence remains insufficient to confirm superiority over conventional methods or long-term clinical benefit. Higher-quality multicenter randomized trials with standardized performance measures are needed to strengthen the evidence base.

## 1. Background

Dental education has undergone significant transformations over the past decades, driven by technological advances and increasing demands for more effective and realistic teaching methods. Traditionally, preclinical training is conducted in laboratories using phantom-head simulators and artificial teeth (typodonts), particularly in disciplines such as operative dentistry [1]. Although widely adopted since their introduction, these models present notable limitations. Among them are the low tactile fidelity, which hinders the accurate simulation of enamel and dentin drilling, the lack of standardization across students’ experiences, and the high consumption of disposable materials, which entails substantial financial costs and environmental concerns [2]. Furthermore, training with typodont teeth is often reduced to manual dexterity exercises, at times reinforcing undesirable habits such as the excessive use of high-speed diamond burs [3,4]. Laboratory infrastructure, frequently constrained by limited space or an insufficient number of workstations, can also restrict the frequency and quality of training. This limitation, however, is not specific to phantom heads.

These limitations directly affect the transition to the clinical environment. Students often struggle with their first procedures on real patients, facing increased levels of anxiety and insecurity [5,6]. The beginning of clinical practice is consistently described as one of the most demanding phases of dental education, not only due to direct patient interaction but also because of the perceived lack of technical preparedness and the pressure to meet clinical performance standards. This scenario underscores the need for pedagogical strategies that promote training experiences more closely aligned with clinical reality, thereby ensuring a gradual and safer transition to professional practice [7].

Within this context, the first digital simulators emerged during the 1990s and 2000s, designed to complement—or in some cases partially replace—traditional training methods. Early prototypes incorporated virtual reality with limited sensory feedback, primarily focused on three-dimensional visualization of teeth and cavities. With advances in computer graphics, robotics, and haptic technology, current virtual reality haptic simulators (VRHS) have been developed [3]. These systems combine immersive three-dimensional environments with tactile, auditory, and vibratory feedback, enabling students not only to visualize but also to feel the resistance differences between enamel, dentin, and carious tissue, thereby simulating clinical procedures such as cavity preparation with greater fidelity [7,8].

Among the most widely used VRHS systems are the Simodont Dental Trainer, Virteasy Dental, and SimToCare, which are implemented in dental schools worldwide [1]. These platforms are reported to enable safe and reproducible practice of procedural tasks, including caries removal and cavity preparation. In addition, they facilitate the development of essential competencies such as hand–eye coordination, fine motor skills, and clinical ergonomics. These tools have also proven valuable for objective assessment, as they allow entire groups of students to perform identical exercises under standardized conditions, thereby improving the consistency of evaluation criteria [1].

It has also been claimed that these VRHS systems, unlike conventional methods, minimize risks associated with the early handling of sharp instruments and provide standardized, objective performance metrics, but there is no current evidence of this. Many of these simulators also integrate artificial intelligence algorithms, which deliver immediate feedback and personalize the learning process by adapting tasks to individual student progress [9].

Nevertheless, the adoption of VRHS faces some challenges. The high initial cost of acquisition and maintenance, the need for adequate technological infrastructure, including high-performance computers and internet connections, and the learning curve required for both students and faculty remain barriers to widespread implementation.

Although recent studies have shown that the use of VRHS enhances procedural accuracy, increases student confidence, reduces technical errors, and fosters the acquisition of essential psychomotor skills in a controlled environment with lower resource consumption [1], gaps persist in the scientific evidence regarding their long-term effectiveness in clinical performance, and the actual contribution of these simulators to preclinical skill acquisition [8,10].

Despite this lack of evidence, dental schools seem eager to acquire and use VRHS systems as students perceive them as an indispensable asset in any modern school.

This systematic review aims to critically examine the role of VRHS in preclinical dental education, with particular focus on cavity preparation. Specifically, it seeks to synthesize the available evidence regarding the impact of their use on psychomotor and technical skill acquisition, explore their effectiveness compared to traditional typodont-based methods, and assess students’ perceptions and acceptance. By addressing these dimensions, this review intends to provide a comprehensive understanding of the applicability and pedagogical value of haptic simulators in contemporary dental education, while also identifying their benefits, limitations, and the knowledge gaps that remain in the literature.

## 2. Methods

### 2.1. Study Sources

A thorough and systematic literature search was performed using the electronic databases PubMed, Scopus, and Cochrane Library to identify relevant studies published between 2015 and 2025. Articles were included if they evaluated the use of VRHS in preclinical dental education, specifically within the context of operative dentistry and cavity preparation training. Additionally, the reference lists of potentially eligible studies were examined to ensure that any relevant articles not captured in the initial database searches were also included. Through this manual search, two additional studies that met the inclusion criteria were identified and incorporated into the final review sample. This systematic review was conducted following the PRISMA (Preferred Reporting Items for Systematic reviews and Meta-Analyses) guidelines [11] and the review protocol was registered in the OSF database (Registration DOI: https://doi.org/10.17605/OSF.IO/JNUFT, accessed on 2 October 2025). Prisma checklist is available in Supplementary Materials (File S1).

### 2.2. Search Strategy

A research question has been formulated following the PICO strategy, which is denoted as P (population), I (intervention), C (comparison), O (outcome).

Population: Undergraduate dental students in the preclinical phase and/or trainees undergoing preclinical training in operative dentistry.

Intervention: Use of haptic simulators (all models/software providing haptic feedback) as a primary or supplementary teaching tool.

Comparison: Conventional teaching methods (mannequins/phantom heads, typodonts, traditional didactic instruction) or other non-haptic technologies.

Outcome: Technical performance/competence and other objective measures of manual skill (e.g., objective performance scores, cavity preparation assessment, quality of the completed work and time to complete tasks, all performed by calibrated examiners). The assessment of these outcomes should be made objectively, using standardized systems or calibrated blinded evaluators.

Below is the defined PICO question:

*Does the integration of virtual reality haptic simulators with traditional typodont-based training improve technical performance in preclinical operative dentistry compared to conventional methods alone?*

The search strategy was developed based on the PICO framework and initially constructed for the PubMed database and adapted for each database consulted. The results from different databases were cross-checked to identify and eliminate duplicates. The search was conducted in September 2025 using the following combination of keywords and Boolean operators: (“dental education [MeSH Terms] OR dental students”) AND (“operative dentistry [MeSH Terms] OR preclinical dentistry”) AND (“haptics [MeSH Terms] OR haptic simulator”). Only studies published within the last 10 years were considered.

Studies were chosen based on the following inclusion criteria: undergraduate dental students or trainees in preclinical operative dentistry; use of virtual reality haptic simulators (VRHS), either as a primary or complementary tool integrated with traditional typodont- or phantom-head-based training; reporting objective measures of technical performance, such as cavity preparation quality, task completion time, error rates, or other quantifiable indicators of manual skill development; original research studies, including randomized controlled trials, quasi-experimental designs, and observational studies; published in English. Exclusion criteria were studies focusing exclusively on clinical training with real patients or postgraduate populations; studies not involving haptic feedback or VR-based simulators; review articles, editorials, commentaries, or conference abstracts without full data; studies not reporting measurable outcomes of technical or manual performance.

The selection of studies was conducted in several steps. First, two independent researchers (PL and HC) scanned relevant articles that fit the study criteria by analyzing the titles and abstracts. The second step involved evaluating the abstracts and non-excluded articles that met the eligibility criteria during the abstract review. The selected articles were read and analyzed individually in relation to the purpose of this study. Disagreements between the two reviewers were resolved by discussion with a third author (MC), who facilitated a consensus through mutual evaluation and deliberation. Cohen’s Kappa test was performed to assess the inter-rater reliability between the two reviewers, quantifying the level of agreement beyond chance. The Rayyan Intelligent Systematic Review Platform was utilized to support the systematic review process [12].

Finally, the relevant information from each selected article was collected and organized into a table highlighting the following aspects: Author/year of publication, Population/sample size, Simulator model, Protocol, Outcomes studied, Measurement tools/scoring and Results.

The methodological quality of the RCT studies was assessed using the Revised Cochrane Risk of Bias Tool for Randomized Trials (RoB 2.0) [13], including specific considerations for cluster-randomized trials, as outlined by the Cochrane Database of Systematic Reviews (2016). This analysis is based on five domains: (1) bias arising from the randomization process; (2) bias due to deviations from intended interventions; (3) bias due to missing outcome data; (4) bias in measurement of the outcome; (5) bias in selection of the reported result [14]. Each domain was evaluated using one of the following judgments: low risk, some concerns, and high risk. The overall judgment for each article was determined by the most prevalent judgment across the domains. Concerning the prospective non-randomized studies, risk of bias was assessed using the ROBINS-I version 2 (variant A) [15], a tool specifically designed to evaluate bias in studies investigating the effects of interventions outside randomized settings. This assessment is based on six domains: (1) risk of bias due to confounding; (2) risk of bias in the classification of interventions; (3) risk of bias in the selection of participants for this study (or for the analysis); (4) risk of bias due to missing data; (5) risk of bias arising from the measurement of the outcome; (6) risk of bias in the selection of the reported result. All evaluations were conducted independently by two reviewers, with any disagreements resolved through consensus.

## 3. Results

### Characteristics of Studies

**Figure 1:**
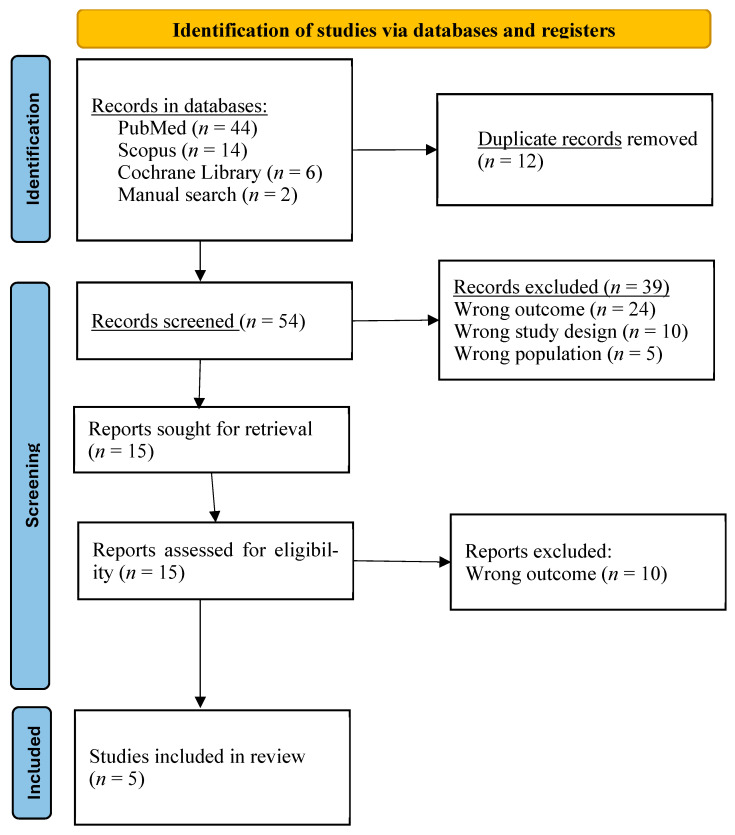
Flow PRISMA diagram of the search strategy and article selection used in the present systematic review.

The initial database search yielded a total of 66 articles, including 44 from PubMed, 14 from Scopus, 6 from Cochrane Library and an additional 2 studies that were identified and included through manual searching of reference lists of potentially eligible articles. After cross-checking and removing 12 duplicates, 54 unique records remained for title and abstract screening. After an initial screening based on title and abstract, 38 articles were excluded for the following reasons: (1) systematic reviews of clinical trials or narrative reviews; (2) studies in population different from dental students; (3) studies that evaluated other procedures rather than operative dentistry. A total of 15 articles were then selected for full reading. Of these, 10 studies were excluded because they evaluated procedures other than cavity preparation in operative dentistry. In the end, five studies were included in this review. The study selection process is described in Figure 1.

The selected studies were analyzed with respect to their methodological quality using the appropriate risk of bias assessment tools. Table 1 presents the risk of bias evaluation for the randomized controlled trials assessed with the RoB 2.0 tool [13], while Table 2 provides the corresponding assessment for the prospective non-randomized studies using the ROBINS-I v2 (variant A) [15].

The methodological quality and risk of bias were assessed using two different tools according to the study design. For the three included randomized controlled trials (RCTs), the RoB 2 tool was applied, and these studies were largely categorized as having an overall low risk of bias. However, in Manav et al. [9] there was a deviation from intended interventions, and therefore it was classified as having a high risk of bias in Domain 2 (D2). Specifically, although no issues related to adherence to the assigned interventions were reported (e.g., no dropouts or non-compliance), an imbalance in practice exposure between groups was identified. The experimental groups received additional simulation training compared to the control group, resulting in a greater overall “dose” of intervention. This unequal exposure was acknowledged by the authors as a potential confounding factor, as it may have influenced the observed outcomes independently of the intervention effect itself. The prospective non-randomized studies were analyzed using the ROBINS-I v2 tool (Variant A) [15]. Both Farag et al. [16] and Rodrigues et al. [17] were identified as having a critical risk of bias in Domain 1 (D1), because there was a risk of confounding factors interfering with the outcome measurements. In both studies students executed the same procedure three times, and regardless of the technology used (Conventional Methods or VR) some improvement was expected. Regarding the remaining domains these studies ranged from low to moderate risk of bias.

**Table 1:** Methodological quality and risk of bias of RCTs assessed with the RoB 2 Tool.

Study	D1	D2	D3	D4	D5	Overall Risk
Manav, E et al., 2025 [9]	Some concerns	High	Low	Low	Low	Low
Daud, A, et al., 2023 [3]	Some concerns	Low	Low	Low	Low	Low
Vincent, M et al., 2020 [18]	Low	Low	Low	Low	Low	Low

**Table 2:** Methodological quality and risk of bias of prospective non-randomized studies assessed with ROBINS-I v2 (Variant A) [15].

Study	D1	D2	D3	D4	D5	D6	Overall Risk
Farag et al., 2021 [16]	Critical	Moderate	Low	Low	Low	Low	Low
Rodrigues, P, et al., 2023 [17]	Critical	Low	Low	Low	Low	Moderate	Low

Table 3 summarizes the main characteristics of the five studies included in this systematic review, assessing the impact of virtual reality (VR) simulators on the development of preclinical skills in dentistry, specifically in cavity preparation (CP).

All investigations involved undergraduate dental students in the preclinical phase of training, typically ranging from the first to the third academic year, with sample sizes varying between 20 and 88 participants. Apart from the study by Manav et al. [9], in which information regarding prior exposure to virtual reality (VR) was not clearly reported, all participants were novices in the use of VR-based simulation. A variety of haptic simulation systems were used, including the Simodont Dental Trainer, VirTeaSy Dental, SIMtoCARE Dente, DENTIFY, and SensAble Omni, primarily for classic Class I cavity preparation tasks. The parameters assessed were similar, commonly included cavity outline and depth, smoothness and orientation of the pulpal floor and cavity walls, internal line angles, marginal ridge integrity, cavity width, and time required for preparation. Evaluation procedures were generally performed by two or more independent and calibrated assessors, with several studies adopting blinded assessment to reduce potential bias. Assessment methods comprised digital grading platforms and standardized scoring systems to evaluate the quality and accuracy of the cavity preparations.

In several studies VR-based training was most consistently associated with gains in efficiency and selected aspects of technical performance. Several papers reported faster task completion with repeated simulator use: Farag et al. [16] observed a reduction in execution time from 46 to 33 min alongside improved psychomotor performance, Rodrigues et al. [17] described a progressive and sustained decrease in instrumentation time consistent with a positive learning curve, and Vincent et al. [18] reported an approximate 30% reduction in task execution time across training sessions.

Improvements in the quality and precision of cavity preparation were also reported, although the effects were not uniform across all evaluated parameters. Farag et al. [16] demonstrated higher mean total cavity preparation scores (9.1 to 12.1) and statistically significant improvements in specific technical features, including pulpal floor smoothness and orientation, alignment of buccal, lingual and mesial walls, and the precision of internal line angles (dihedral and trihedral). Vincent et al. [18] similarly found enhanced drilling accuracy and reduced iatrogenic damage during cavity preparation, suggesting improved hand–eye coordination supported by the simulator’s computerized visual feedback, when subsequently assessed in an analogue setting using plastic teeth. VR-trained students performed comparably to or better than those trained exclusively with conventional methods across most parameters, including cavity contour and depth. In contrast, Manav et al. [9], when combining VR simulators with tactile feedback and layered tooth models (Caviprep), found that depth control accuracy significantly improved (*p* = 0.001), while no significant differences were observed for other parameters such as cavity contour, pulpal floor smoothness, internal line angles, marginal ridge integrity, or retention form (*p* > 0.05). In the same study, overall cavity preparation quality scores did not differ between groups (*p* = 0.715). The combined VR-plus-layered-model approach did not confer an additive advantage over each modality used in isolation, a finding the authors linked to potential cognitive overload in novices and the short training period (two days). Taken together, these results suggest that VR tools may be particularly effective for enhancing depth control and selected fine motor components early in training, while broader improvements across all preparation criteria are less consistently demonstrated.

Students’ perceptions converged on VR as a valuable, motivating adjunct to conventional preclinical training rather than a replacement. Daud et al. [3] reported that students perceived virtual reality haptic simulators (VRHS) as effective for improving manual dexterity and psychomotor skills, highlighting the ability to practice repeatedly in a safe environment without irreversible errors and improved visualization of dental anatomy and tissue layers. Rodrigues et al. [17] similarly noted positive qualitative feedback, with students reporting increased interest in operative dentistry, greater awareness of applied manual force, and support for developing manual dexterity and three-dimensional visualization. Importantly, participants also pointed to practical limitations that currently constrain full substitution of mannequin-based training, including tactile feedback fidelity, handpiece ergonomics, and the absence of simulated water spray [3]. From a pedagogical perspective, there was strong consensus among students regarding the value of VRHS for enhancing manual dexterity and engagement in learning. While Daud et al. [3] emphasized that students viewed VRHS as a valuable adjunct to standard training, they also highlighted limitations related to hardware and software fidelity. Additionally, Rodrigues et al. [17] reported that learners valued the simulator’s performance metrics as more objective than traditional plastic model assessment, particularly prolonged contact alerts that heightened awareness of iatrogenic risks such as pulp overheating.

The evidence suggests that although VRHS do not consistently outperform traditional methods in terms of technical quality, they represent an effective complementary tool that enhances learning curves, procedural efficiency, and student engagement in preclinical operative dentistry.

## 4. Discussion

The findings of this systematic review indicate that the integration of virtual reality haptic simulators (VRHS) with conventional typodont-based training enhances psychomotor skill acquisition and technical performance in preclinical operative dentistry. These outcomes align with previous research [8,10] suggesting that immersive, multisensory learning environments improve motor coordination, spatial perception, and procedural accuracy in dental training.

The hybrid model, combining virtual and conventional methods, appears particularly effective in bridging the gap between theoretical learning and clinical application. This integration not only allows for repetitive and standardized practice but also provides real-time feedback and objective performance assessment—advantages not easily achieved in traditional laboratory settings. For instance, studies by Manav et al. [9] and Farag et al. [16] demonstrated that the use of VRHS reduced task completion time while maintaining or improving precision, reflecting a more efficient learning curve. Such findings reinforce the pedagogical value of simulation-based training in promoting both competence and confidence prior to clinical exposure.

Beyond the psychomotor benefits, several authors have highlighted the cognitive and affective dimensions of VRHS-based training. Studies by Truchetto et al. [19] and Al-Saud et al. [20] demonstrated that immersive learning environments improve spatial awareness and procedural reasoning, leading to greater retention of operative concepts and reduced cognitive load during manual execution. This suggests that haptic simulators contribute not only to motor refinement but also to higher-order learning processes, fostering a deeper integration between theory and practice. Moreover, immersive VR promotes engagement and reduces performance anxiety, a factor of considerable relevance in early preclinical stages where students frequently report stress and fear of error.

However, while VRHS training supports skill enhancement, it should not be regarded as a complete substitute for traditional preclinical exercises. Studies such as Daud et al. [3] emphasized that mannequin- or typodont-based practice remains essential for developing tactile sensitivity and contextual understanding of clinical ergonomics. Haptic simulators may, therefore, serve best as a complementary tool within a blended curriculum, where digital and manual methods are integrated to optimize student outcomes.

The consistent positive feedback from students regarding engagement, motivation, and self-perceived preparedness also highlights the potential of VRHS to enhance the overall learning experience. The gamified and interactive nature of these tools can foster active learning and continuous self-assessment, which are critical components of competency-based education. Nonetheless, the high cost of acquisition and maintenance, the requirement for specialized infrastructure, and the need for faculty training remain major barriers to large-scale implementation.

The main limitation of this review is the heterogeneity among studies in simulator type, evaluation metrics, and training duration. This heterogeneity limits the comparability of results and underscores the need for standardized methodologies.

VRHS integration promotes the ecological and economic sustainability of institutions which is an important dimension. Traditional typodont-based training requires extensive use of plastic teeth, burs, and other consumables, leading to significant financial and environmental costs. By contrast, VR systems enable repetitive practice without material waste, arguably aligning dental education with sustainable development principles and institutional green policies. Although the initial cost of VRHS acquisition is high, cost–benefit analyses indicate that long-term savings from reduced consumables, maintenance, contact hours and space requirements can offset initial investment, particularly when devices are shared across multiple courses or faculties [1,8,21,22].

Future research should focus on longitudinal and multicenter studies to assess the long-term impact of VRHS training on clinical performance and patient outcomes. Establishing validated assessment frameworks and consensus on performance metrics will be essential to strengthen the evidence base and facilitate curricular integration. Furthermore, exploring the role of artificial intelligence within haptic simulators could offer new possibilities for adaptive, personalized feedback and automated skill assessment.

## 5. Conclusions

This systematic review suggests that virtual reality haptic simulators (VRHS) are effective tools for enhancing early psychomotor skill development and procedural efficiency in preclinical dental education. However, current evidence does not fully support their superiority over conventional methods, indicating that they should be used as complementary rather than a replacement.

From an educational standpoint, VRHS enable standardized, feedback-driven training that promotes skill acquisition and student confidence. Clinically, they may support a safer transition to patient care by allowing students to develop competencies in a controlled environment. Effective implementation requires structured integration into dental curricula, including clear guidance on timing, combination with traditional methods, and assessment strategies. Future research should focus on long-term clinical outcomes and the development of standardized guidelines to optimize the use of VRHS in dental education.

## Figures and Tables

**Table 3:** Relevant data gathered from the retrieved studies. All studies were experimentally either randomized or non-randomized (shaded) and all students were novices in the use of VR except in Manav et al. [9], where information on previous VR use is not clear.

Author/Year	Population/Sample Size	Simulator Model	Protocol	Outcomes Studied	Measurement Tools/Scoring	Results
Daud, A, et al., 2023 [3]	3rd-year dental students (*n* = 23)	SIMtoCARE Dente (class I)	Group 1: VR → CM Group 2: CM → VR	Cavity Outline Depth of Cavity	CM was graded digitally on Blackboard	Novice dental students generally perceive the VRHS tool as useful for enhancing manual dexterity. It should be employed to support, rather than replace, CM.
Farag et al., 2021 [16]	3rd-year dental students (*n* = 21)	Simodont dental trainer	CM → 4 week training in VR → CM	Cavity Outline Pulpal Floor (smoothness and direction) Internal Line Angles Wall Direction (buccal and lingual) Time spent in CP	CM assessed by 2 calibrated evaluators	Novice dental students generally perceive VRHS as useful tools for enhancing manual dexterity. Time for CP decreases and quality of CP increases with VRHS training. VRHS should be employed to support, rather than replace, CM.
Manav, E et al., 2025 [9]	2nd-semester dental students (*n* = 48)	VirTeaSy Dental (class I)	Group 1: CM (model) → VR Group 2: VR only Group 3: CM (model) only Group 4: CM on natural teeth	Cavity Outline Pulpal Floor Internal Line Angles Depth of Cavity Marginal Ridge Wall Direction (buccal and lingual) Cavity Width	CM assessed by 3 calibrated evaluators	Integration of VR simulators and models enhances depth control during CP in early dental training. These tools may provide benefits in developing manual skills essential for clinical competence.
Rodrigues, P, et al., 2023 [17]	3rd-year dental students (*n* = 20)	DENTIFY (Class I e Class II)	CM → VR → CM	Time spent in CP	CM was scanned for assessment	Novice dental students generally perceive VRHS as useful tools for enhancing manual dexterity. Time for CP decreases and quality of CP increases with VRHS training. VRHS should be employed to support, rather than replace, CM.
Vincent, M et al., 2020 [18]	1st-year dental students (*n* = 88)	Virteasy (Class I)	Group 1 VR → CM. Group 2: CM	Cavity Outline Depth of Cavity Pulpal Floor smoothness Time spent in CP % of target tissue removed	CM assessed by 2 calibrated evaluators M assessed by 2 calibrated evaluators	Novice dental students generally perceive VRHS as useful tools for enhancing manual dexterity. Time for CP decreases but quality of CP is not significantly different when compared to the CM. VRHS should be employed to support, rather than replace, CM.

VR—virtual reality; CM—conventional methods; CP—cavities preparation; VRHS—virtual reality haptic simulators. Arrows (→) indicate sequential events.

## Data Availability

The original contributions presented in this study are included in the article and Supplementary Materials. Further inquiries can be directed to the corresponding author.

## Supplementary Materials

The following supporting information can be downloaded at https://www.mdpi.com/article/10.3390/dj14050314/s1: File S1: PRISMA 2020 checklist.
